# Exposure to Chlorinated Biphenyls Causes Polymorphonucleocytes to Induce Progenitor Cell Toxicity in Culture

**DOI:** 10.3390/ijerph2006030003

**Published:** 2006-03-31

**Authors:** Dwayne A. Hill, Carroll T. Reese, Dwane Clarke, Tanika V. Martin

**Affiliations:** 1 Department of Biology, School of Computer, Mathematical and Natural Sciences, Morgan State University, Baltimore, Maryland 21251

**Keywords:** Polychlorinated Biphenyls, Aroclor, Neutrophils, Polymorphonucleocytes, Progenitor cells, Toxicity

## Abstract

Progenitor cells (PC) are the precursors for many developmental structures and are sensitive to a variety of toxic agents including the environmental contaminants, polychlorinated biphenyls (PCBs). The mechanism(s) that contributes to the development of PCB-induced progenitor cell-related fetotoxicities are not completely understood. However, several studies have demonstrated an important role for neutrophils (polymorphonucleocytes) in the development of PCB induced toxicities. Our recent findings have indicated that conditioned medium collected from PC (CMPC) exposed to a single dose of the PCB mixture, Aroclor 1248, can activate isolated neutrophil populations. Because of our recent findings, this study was conducted to determine if conditioned medium from PC treated with a PCB mixture causes neutrophils to injure PC in culture. Isolated PC were cultured and treated with different concentrations of Aroclor 1248 for 24 hours. The resulting PC-derived conditioned media was collected and its affect on neutrophil activity was analyzed. Conditioned medium from PC treated with Aroclor 1248 was chemotactic for neutrophils. The conditioned medium from Aroclor 1248 treated-PC also stimulated neutrophils to release super oxide anion, cathepsin G and elastase into culture medium. Furthermore, the conditioned medium from Aroclor 1248 treated- PC was able to stimulate neutrophils to cause progenitor cell toxicity in co-cultures. The conditioned medium from Aroclor 1248 treated-PC was not toxic to individual neutrophil cultures or PC cultures. Moreover, the addition of a protease inhibitor to the co-cultures containing neutrophils and PC, afforded protection against neutrophil-induced cytotoxicity of PC. These data suggest that a PCB mixture can cause progenitor cells to produce a factor(s) that activates neutrophils and stimulates them to damage PC populations in culture.

## Introduction

Polychlorinated biphenyls (e.g. Aroclor 1248) are persistent environmental contaminants [3, 4]. In adult mammals, exposure to PCBs can result in a variety of systemic toxicities with severe inflammation, hepato-degeneration, neurological alterations, skin disorders and GI-tract abnormalities among the more common [1, 2]. In addition, these environmental contaminants have demonstrated teratogenic activity [3, 4, 5] that can be manifested as abnormal cranial/facial structures and aberrant neuronal development [3, 4, 5]. Certain progenitor cell populations, such as the neural crest parenchyma, are the critical for the development of various fetal/embryonic structures [14, 15]. These progenitors are of epithelial origin, maintain a significant degree of auto- and para-cellular regulation, and are highly migratory [16]. Certain populations of epithelial cells have been shown to produce factors that regulate neutrophil activity [9, 10, 19, 21] Studies have demonstrated that the inflammatory response of neutrophils contributes to the development and propagation of PCB-induced toxicity within adult subjects [8, 9]. Whether neutrophils are involved in the teratogenic affects of PCBs is unclear and requires further elucidation. Accordingly, we designed this study to determine if PC, in response to PCB exposure, produces a factor(s) that can cause the chemotaxis of neutrophils and stimulate them to release cytotoxic proteases and other toxic agents that could cause PC damage.

## Materials and Methods

### Isolation of Progenitor Cells

Female Sprague-Dawley rats (CD-Crl:CD(SD), Harlan Labs. Inc. Fredrick, Maryland) were obtained on day five of pregnancy and allowed to acclimate for 4 days. Rat conceptuses were explanted on gestational day 10 as described by Ambroso and Harris (1994). The explanted conceptuses were then cultured (24 hours) in medium containing 33% heat-inactivated pregnant rat serum, 66% Hank’s Balanced Salt Solution (HBSS), and 1% gentamycin (Sigma chemical, St. Louis, MO). The conceptuses were then removed from the culture medium, rinsed with HBSS and placed in Tyrode’s dissecting buffer (Sigma chemical, St. Louis, MO) for the isolation of neural crest cells (progenitor cell population). This progenitor cell population was isolated from the embryos by initially removing the ectoplacental cone, visceral yolk sac, and the amnion to expose the intact embryo. The neural tubes were then surgically dissected from the embryo and placed into Tyrode’s dissecting medium containing 0.025% trypsin. The somites and excess tissue were removed so that the neural tube was completely exposed. The isolated neural tubes were then rinsed with Tyrod’s medium void of trypsin and transferred into a Sigma 12 well culture plate coated with 0.75 mLs collagen matrix. Each well contained 2 neural tubes and 3 mLs Dulbecco’s modified eagles medium (DMEM) (Sigma chemical) supplemented with 10% chick embryo extract (Sigma chemical), 10% fetal calf ser um and 1% gentamycin/pen/strep (Sigma chemical). After a 24 hour incubation period, the progenitor cells migrated away from the neural tube and began colony expansion. The neural tubes were removed from the culture at this time. After a 24 hour expansion period, the cell cultures were washed. The viable cells remained attached to the plate while the other cell types were lifted due to the incompatible culture conditions. Fresh medium was added and viability was determined by trypan blue exclusion. Progenitor cell (PC) cultures were maintained using DMEM containing the respective supplements previously mentioned. Cytospins and HNK staining was conducted to verify PC populations.

### Isolation of Neutrophils

Neutrophils (polymorphonucleocytes) were isolated from female Sprague-Dawley rats (Charles River Laboratories, 250–300 grams) by glycogen elicitation as described by Dahm et al. [8]. Thirty mLs of 1% glycogen (sterile saline vehicle) were injected into the peritoneum of anesthetized rats. After a 4-hour period, the rats were sedated and decapitated. Neutrophils were isolated via a rinse of the peritoneum with 30 mLs phosphate-buffered saline (PBS) containing 1 unit heparin/mL. The neutrophil-containing solution was then filtered through standard 4 × 4 gauze pads (Sigma chemical, St. Louis, MO) and centrifuged (Allegra 6R, Beckman) for 7 minutes/500 × g. Red blood cells contaminating the cell preparation were lysed with NH_4_Cl (0.2M). Next, the isolated neutrophils were washed twice with PBS, resuspended in Williams’ Medium-E containing 1% gentamycin and cultured in Falcon six-well polystyrene plates at a density of 1 × 10^6^ cells/mL. Purity of neutrophil isolations was routinely > 95%, and viability was > 98% (lactate dehydrogenase release and trypan blue exclusion, respectively).

### Exposure of Isolated Progenitor Cells to Aroclor

Either Aroclor 1248 (standard PCB mixture) or the respective vehicle (naive Williams’ Medium E) was added to isolated PC (5 × 10^6^ cells/mL) to obtain a final concentration of 0, 6, 12, 25 or 50 μM and incubated for 24 hours. PC viability was assessed by eosin Y uptake analysis.

### Preparation of Conditioned Medium from PC Treated with Aroclor (CMPC-Aroclor)

PC were plated (1 × 10^6^ cells) in Falcon 3046, six-well culture plates and allowed to adhere for 3–4 hours before treatment with aroclor 1248 at a final concentration of either 6, 12, 25 or 50 μM or its vehicle for 24 hours. At this time, the conditioned medium was collected, centrifuged at room temperature for 15 minutes at 120 × g to remove cellular debris, and then stored at −20°C. To indicate PC toxicity, hyaluronan levels in the culture medium were measured as an indicator of toxicity. Hyaluronan levels in the culture medium were measured as an indicator of PC toxicity. After the incubation period, culture medium was analyzed for hyaluronan levels as a specific marker of PC toxicity. The hyaluronan levels were measured using a HA-ELISA kit from Echelon Inc.

### Treatment of Isolated Neutrophils with Conditioned Medium Derived from PC

Either CMPC, CMPC-Aroclor or vehicle (naïve Williams’ Medium E) was added (1:1; v:v) to isolated neutrophils and incubated for 24 hours at 37°C. After the incubation period, culture medium from isolated neutrophils was analyzed for lactate dehydrogenase activity (LDH) to indicate neutrophil toxicity.

### Chemotaxis of Neutrophils to Conditioned Medium Derived from PC

A two-chambered chemotaxis system was used to determine if the conditioned medium derived from PC was chemotactic for neutrophils. The upper chamber, a Falcon 3181 Cell Culture Insert (Becton Dickinson, Franklin Lakes, NJ), was separated from the lower chamber, a Falcon 3043 Multiwell Culture Plate (Becton Dickinson, Franklin Lakes, NJ), by a 3 μm cyclopore polyethylene terephthalate membrane located at the base of the insert. Neutrophils (1.5 × 10^6^ cells) were placed in the upper chamber, and conditioned medium (1.0 mL) from PC was placed in the lower chamber. In response to a chemotactic stimulus, neutrophils migrate through the membrane to the lower chamber. One hour after both chambers were loaded, the degree of chemotaxis was determined by counting neutrophils present in the lower chamber. N-Formyl-methionyl-leucyl-phenylalanine (FMLP [10 nM]) was used as the positive control stimulus for chemotaxis, and chemotaxis was expressed as % of this positive control.

### Isolation and Measurement of Protease Activity in Culture Medium Collected from Neutrophils Treated with CMPC-Aroclor

Neutrophils (2 × 10^6^ cells/mL) were treated with CMPCP, naive medium, or CMNC for 24 hours. After this incubation period the neutrophil-derived conditioned medium was collected for analysis of protease activity. Cathepsin-G and elastase were isolated from the culture medium. Briefly, Cathepsin-G and elastase were isolated by methods previously established [9]. Briefly, proteases were precipitated from the neutrophil-derived conditioned medium using ammonium sulfate (65% saturation). The precipitate was collected by centrifugation (15,000 × g/30 minutes). The resulting pellet was resuspended in 8 mL of sodium acetate buffer (50 mM sodium acetate containing 1M sodium chloride, pH 4.0), passed through Spectra/Por-1 (6–8 KD cutoff) or Spectra/Por 4 (12–14 KD cutoff) columns (Waters Inc., Milford, MA) and eluted with sodium-acetate buffer for 6 hours. Once isolated, protease activity was measured spectrophotometrically (405 nm) using N-succinyl-Ala-Ala-Pro-Phe-p-nitroanilide and N-methoxysuccinyl-Ala-Ala-Pro-Val-p-nitroanilide as substrates, respectively [17].

### Measurement of Superoxide Production by Neutrophils Treated with CMPC-Aroclor

Neutrophils (2 × 10^6^ cells/mL) were treated with CMPC-Aroclor, CMPC or naive medium, for 24 hours. After this incubation period the neutrophil-derived superoxide activity was analyzed. Briefly, superoxide anion activity was determined by the reduction of cytochrome c (100 micromolar; horse heart, type III, SigmaAldrich, St. Louis, Mo), using kinetic changes in spectrophotometric readings (absorbance - 550 nm). The rapid reduction of cytochrome C by neutrophil-derived superoxide was calculated as nmol of superoxide formed via extinction coefficient of 21 × 10^3^ M-1 cm-1 but expressed as percentage positive control (FMLP; fMet-Leu-Phe). The production of superoxide in response to FMLP after was completed after a 25 minute incubation and ended via superoxide dismutase (30 units/ml; SigmaAldrich, St. Louis, Mo) administration.

### Treatment and Analysis of Neutrophil-Induced PC Toxicity

Either CMPC, CMPC-Aroclor or vehicle (naïve Williams’ Medium E) was added (1:1; v:v) to co-cultures containing neutrophils (2 × 10^6^ cell/mL) and PC (5 × 10^6^ cells/mL). The co-cultures were incubated for 24 hours at 37°C. After the incubation period, culture medium was analyzed for hyaluronan levels as a specific marker of PC toxicity. The hyaluronan levels were measured using a HA-ELISA kit from Echelon Inc.

### Inhibition of Neutrophil-Induced PC Toxicity

Superoxide dismutase (SOD; 1000 units/mL) and trypsin-chymotrypsin inhibitor (TCI 10 ng/mL; protease inhibitor) was added to neutrophil/PC co-cultures (2 × 10^6^ cells/mL, and 5 × 10^6^ cells/mL, respectively) with or without conditioned medium derived from PC treated with Aroclor. The co-cultures were incubated for 24 hours at 37°C. After the incubation period, culture medium was analyzed (ELISA) for hyaluronan levels as a specific marker of PC toxicity.

## Statistical Analysis

Results are expressed as mean + SEM. Data were analyzed by analysis of variance (ANOVA), and individual means were compared using Dunnett’s test. Appropriate transformations were performed on data for which variances were not homogeneous. An *N* = 9 was used for all study groups and conditioned medium derivations unless otherwise indicated. The criterion for significance was p < 0.05.

## Results

### Viability of PC after Exposure to Aroclor

Initially experiments were conducted to determine if treatment of progenitor cells (PC) with different concentrations of Aroclor caused an increase in PC lethality. Thus, PC were isolated and incubated with either Aroclor_(6, 12, 25, or 50 uM)_ for 24 hrs. The subsequent data indicated that neither concentration of Aroclor resulted in statistically significant changes in PC viability (Table 1).

### Effect of Aroclor on PC Cytotoxicity

Studies were then conducted to determine if the same concentrations of Aroclor that were non-lethal to PC caused an increase in cytotoxicity. Therefore, isolated PC were incubated with Aroclor_(6, 12, 25, or 50 μM)_ for 24 hrs. After this incubation period the levels of hyaluronan released in the culture medium were determined as an indication of PC toxicity. The results indicated that the higher concentrations of Aroclor (25 and 50 μM) caused a significant increase in PC cytotoxicity when compared to control groups. The lower concentrations of Aroclor (6 and 12 μM) did not cause an increase in hyaluronan release (Table 2). The data from Tables 1 and 2 suggested that the aforementioned concentrations of Aroclor caused PC injury without direct PC lethality.

### Induction of Neutrophil Toxicity by CMPC-Aroclor

Studies were then conducted to determine if the conditioned medium from progenitor cells treated with Aroclor (CMPC-Aroclor_(6, 12, 25, or 50 μM)_) altered neutrophil viability. In order to investigate this parameter, CMPC-Aroclor_(6, 12, 25, or 50 μM)_ was collected and then added to isolated neutrophil cultures. After a 24 hour incubation, neutrophil viability was determined using trypan blue exclusion and eosin Y uptake assays. When cells are non-viable, trypan blue is easily distributed to the intracellular domain of the cells and exhibit a blue color phase. In addition, when cells are non-viable they will internalize eosin Y and a redish color phase can be observed optically. The results indicated that PC-derived conditioned medium did not cause any overt neutrophil toxicity (Table 3).

### Ability of CMPC-Aroclor to Stimulate Neutrophil Chemotaxis

In order to determine if CMPC-Aroclor was able to induce neutrophil migration, a two-chambered vertical chemotaxis system was used. Neutrophils (1.5 × 10^6^ cells) were placed in the upper chamber, and CMPC-Aroclor was placed in the lower chamber. In response to the potential stimulus (CMPC-Aroclor), neutrophils migrated through the membrane barrier (separating the upper and lower chambers) to the lower chamber.

The results indicated that the CM derived from PC treated with 25 or 50 μM Aroclor was able to stimulate chemotaxis. In addition, the remaining derivations of CMPC-Aroclor were not effective in stimulating neutrophil chemotaxis (Table 4). These results suggested that CMPC-Aroclor can stimulate neutrophil activity and chemotaxis is just one function important for neutrophil-induced cytotoxicity. Therefore, we investigated additional parameters important for neutrophil cytotoxic activity with a focus on the release of neutrophil derived proteases and superoxide production.

### Induction of Neutrophil-Derived Protease Release by CMPC-Aroclor

To determine if CMPC-Aroclor can influence the release of specific proteases, isolated neutrophils were incubated with CMPC-Aroclor_(6, 12, 25, or 50 μM)_ for 24 hrs. After this incubation period, the culture medium was collected for the measurement of cathepsin-G and elastase levels (Table 5). The results indicated that CMPC-Aroclor, derived from 25 or 50 μM, caused a significant increase in the release of cathepsin-G and elastase from neutrophils. All other derivations of CMPC were non-stimulatory.

### Induction of Neutrophil-Derived Superoxide Production by CMPC-Aroclor

Studies were also conducted to determine if CMPC-Aroclor could stimulate superoxide production from isolated neutrophils. In order to analyze this parameter neutrophils were incubated with CMPC-Aroclor_(6, 12, 25, or 50 μM)_ for 24 hrs. After this incubation period, the production of superoxide anion by this cell population was analyzed using a cytochrome C reduction assay (Table 6). Table 6 illustrates the ability of the CMPC-Aroclor, derived from 25 or 50 μM, to cause a significant increase in the production of neutrophil-derived superoxide anion. All other derivations of CMPC were non-stimulatory.

### Ability of CMPC-Aroclor to Cause PC Cytotoxicity in Co-Cultures of Neutrophils and PC

In order to determine the ability of CMPC-Aroclor to stimulate neutrophil-induced cytotoxicity in PC, Isolated neutrophils were co-cultured with PC in the presence or absence of CMPC-Aroclor. These studies indicated that neither naïve CMPC or CMPC derived from 6 or 12 μM of Aroclor were able to stimulate neutrophils to damage PC in co-culture. Conversely, CMPC derived from 25 or 50 μM of Aroclor was able to stimulate a significant degree of neutrophil-dependant PC cytotoxicity (Table 7). Elevated levels of hyaluronan release into the co-culture medium indicated the increase in PC cytotoxicity. Simultaneous to the PC cytotoxicity, the levels of cathepsin G, elastase and superoxide were increased. Therefore it is conceivable that Co-culture studies in the presence of specific cathepsin G, elastase and/or superoxide inhibitors will provide further insight on the direct involvement of each in the induction of cytotoxicity. Therefore to provide further insight on the direct involvement of cathepsin G, elastase and superoxide in the induction of cytotoxicity, we performed co-culture studies in the presence of specific inhibitors for these markers.

### Ability of Trypsin-Chymotrypsin Inhibitor (TCI) and Superoxide Dismutase (SOD) to Protect Against PC Cytotoxicity Induced by Neutrophils Stimulated with CMPC-Aroclor

In order to determine the cytotoxic role of neutrophil-derived substances (cathepsin G, elastase and/or superoxide) in this model of inflammatory cell-mediated PC injury, TCI and SOD were added to neutrophil/PC co-cultures containing CMPC-Aroclor (Table 8). The results indicated that TCI and SOD, alone, are able to reduce the degree of PC cytotoxicity by approximately 50% (Table 8). Moreover, co-administration of TCI/SOD provides complete protection against PC injury induced by cathepsin G, elastase, and/or superoxide (Table 8; this inhibitory/protective activity was indicated by a significant decrease in the elevations of hyaluronan in the presence of the specific inhibitors). These results also indicated that the neutrophil-derived proteases and superoxide contribute equally to the injury of PC in co-cultures.

## Discussion

In adults, neutrophils have been shown to play a role in a variety of cytotoxicities by responding to factors released by tissue associated cell populations that have been exposed to contaminants and/or pollutants [10, 11, 18, 19]. It is unclear whether a similar phenomenon and/or mechanism occur during the development or progression of certain fetal and/or embryonic toxicities. However, the potential mechanism(s) that facilitate these abnormal events require further clarification. The PC population of neural crest paranchyma are essential progenitors for the development of certain structures contained in different organ systems [12, 13, 14, 15, 16].

The present study has shown that in response to exposure to the PCB mixture, Aroclor 1248, PC produce a factor(s) that causes neutrophils to chemotax and release cytotoxic proteases and reactive oxygen species. These observations are indicative of a potential pathway involving immuno-effector cells in the development or progression of fetal/embryonic toxicity.

It is important to emphasize that little research, has been documented concerning the involvement of neutrophils in the genesis and propagation of progenitor cell toxicity. For example, studies conducted by Ganey (2005) have indicated that PCBs, depending on their structural orientation, are able to influence neutrophil activity. This influence can result in a variety of tissue specific cytotoxicities with liver and lung being very prominent. However, this study, which provides valuable information, does not include PCB-induced alterations in parenchyma and parenchyma-derived factors regulating neutrophil activity. In addition, a significant void of documented information also exists concerning the ability of PC (in response to a toxic challenge) to produce a factor(s) that can regulate neutrophil activity. However, we will endeavor to address the results and discuss them as they correlate with the relevant body of literature that does exist to date.

The progenitor population of neural crest cells is critical for the development of the cranio-facial structures. These cells have been shown to be very susceptible to the toxicities of various environmental contaminants [6, 7]. Since PCBs have been documented to cause alterations in feto-cranial development, it is possible that PCBs could target this progenitor cell population for the induction of fetotoxicity [3, 4]. Studies have demon strated that the inflammatory response of neutrophils contributes to the development and propagation of PCB-induced toxicity within adult subjects [8, 9]. Whether neutrophils are involved in the teratogenic affects of PCBs is unclear and requires further elucidation. Accordingly, this study was designed to determine if PCB mixtures could cause certain progenitor cells to produce a factor(s) that activates neutrophils and stimulates them to damage progenitor cell populations.

The present study indicated that Aroclor 1248 was not lethal to PC at concentrations of 6 and 50 μM (Table 1). This observation provided evidence that low concentrations of PCBs did not cause PC death *in vitro*. This observation was very relevant since the cellular uptake, distribution kinetics, and toxic index of this contaminant *in vivo* is a function of the exposure dose ranging between middle and high uM concentrations [4, 6]

Conditioned medium from PC treated with a minimum of 25 μM Aroclor 1248 was shown to be chemotactic for neutrophils in comparison to control chemotactic agents (Table 4). However, the degree of neutrophil chemotaxis was markedly augmented when PC-derived conditioned medium was generated by ≥ 50 uM Aroclor 1248. These observations suggest that the production of CMPC-Aroclor that is chemotactic for neutrophils, *in vitro,* is dose dependent.

The present study has also demonstrated that CMPC-Aroclor has the ability to cause neutrophils to produce and release superoxide anion *in vitro* (Table 6). This observation demonstrated the ability of CMPC-Aroclor to activate neutrophils because radical species production and release is a fundamental and widely accepted marker of neutrophil activation.

In addition, CMPC-Aroclor causes neutrophils to release the proteases, cathepsin G and elastase *in vitro* (Table 5). This observation demonstrated the ability of this PC-derived conditioned medium to directly activate neutrophils. Others have also demonstrated the ability of contaminants to cause epithelium/endothelium to release substances that regulate neutrophil activation. Investigations by Holle (2005) [25] demonstrated that verocytotoxin causes endothelium to release chemokine-like factors that enhanced neutrophil migration/activation that could influence endothelial homeostasis [25] in the renal domain. In another study, Gordon (2003) [26] demonstrated that repeated exposures to salbutamol causes the increased release of CXCL8 which has chemokine-like activity on neutrophils causing them to undergo chemotaxis and activation [26]. This activation could result in compromised pulmonary responses. It is encouraging to discover that the results from our current study have remained consistent with the patterns of activity/response models presented in the literature.

Although all the specific mechanisms facilitating cytotoxicity in our model design are not fully understood, there is an emerging body of evidence that the ideology of inflammatory response effectors (neutrophils) and their regulation/modulation play an important role in the onset of many tissue organ specific cytotoxicities.

Furthermore, this study showed the ability of CMPC-Aroclor to stimulate neutrophils to damage PC in co-culture using neutrophil-derived proteases and reactive oxygen species (Table 7 and 8). These results also demonstrated that both neutrophil-derived proteases and reactive oxygen species play an equivalent role in the degree of neutrophil-mediated injury to isolated PC in co-culture (Table 7 and 8).

Our studies suggest that an undetermined regulatory factor is present in the PC-derived conditioned medium. However, certain epithelial cell populations have been shown to produce neutrophil-regulatory factors in response to a toxic challenge. For example, Schmouder (1993) and Kayama (1995) [24, 18] demonstrated that certain nephrotoxicants cause renal epithelial cells to release factors (e.g. cytokin e-induced neutrophil chemoattractant [CINC], monocyte chemotactic peptide-1 [MCP-1]) that cause n eutrophil chemotaxis and activation. In addition, studies conducted by Driscoll (1994) [19] demonstrated that certain toxic agents stimulate epithelial cells lining the small airways of the respiratory tract of rodents to produce chemokines (e.g. macrophage inflammatory protein [MIP], cytokine-induced neutrophil chemoattractant [CINC]) capable of inducing neutrophil migration and activation. Beck-Schimmer (2005) [27] also demonstrated that lung epithelial cells produce macrophage inflammatory protein [MIP] that stimulates neutrophil infiltration in response to toxic challenge. In view of the present study and the aforementioned observations, it is possible that PC respond to certain toxic agents similarly by producing substances or factors, in response to an environmental contaminant, that are chemotactic for neutrophils and stimulates them to release cytotoxic factors. Nevertheless, the production of other factors (e.g. interleukins, interferons) that modulate neutrophil activity or recruit other immuno-effector cells cannot be completely ruled out [17, 18, 22].

In conclusion, the current study indicates that in response to Aroclor 1248, PC produce an unknown factor that was chemotactic for neutrophils and caused them to release the cytotoxic proteases, cathepsin G and elastase, and superoxide anion which subsequently induced PC damage *in vitro*. Additionally, the presence of the protease inhibitor, TCI, and the superoxide inhibitor, SOD, resulted in complete protection against neutrophil-mediated damage as indicated by PC-derived hyaluronan release into co-culture medium. In the immediate future, efforts will be directed towards the identification of the PC-derived factor(s) that is capable of regulating neutrophil activation in this model. This information will be important for the continued elucidation of the mechanisms involved in this potential toxicity pathway.

## Figures and Tables

**Table 1:** *Effect of Aroclor on Progenitor Cell Viabiliy:* Isolated PC (5 X 10^6^ cells/mL) was incubated with Aroclor_(6, 12, 25 or 50 μM)_ or vehicle for 24 hrs. After this incubation, PC was washed and viability was assessed by eosin Y uptake analysis. Aroclor_(X)_ = PCB mixture, Aroclor, where (X) is the concentration of Aroclor; Values represent mean ± S.E.M.; a, indicates values that are significant (p≤0.05).

*Additions to Progenitor Cell Cultures*	*Progenitor Cell Viability (%)*
Naive	97 ± 4.1^a^
Vehicle	94 ± 5.4^a^
Aroclor_6_	91 ± 4.4^a^
Aroclor_12_	93 ± 2.3^a^
Aroclor_25_	95 ± 5.1^a^
Aroclor_50_	94 ± 8.2^a^

**Table 2:** *Cytotoxicity of Aroclor on Progenitor Cell Cultures:* Isolated PC (5 X 10^6^ cells/mL) was incubated with Aroclor _(6, 12, 25 or 50 μM)_ or vehicle for 24 hrs. After this incubation, PC was washed and the analysis of hyaluronan release was used as the endpoint for the determination of cytotoxicity. Aroclor_(X)_ = PCB mixture, Aroclor, where (X) is the concentration of Aroclor; Values represent mean ± S.E.M.; where either a, b or c indicate values that are significantly (p≤0.05) different from each other.

*Additions to Progenitor Cell Cultures*	*Release of Hyaluronin (ng/ml) by Progenitor Cells*
Naive	7 ± 4.1^a^
Vehicle	9 ± 5.4^a^
Aroclor_6_	8 ± 4.4^a^
Aroclor_12_	9 ± 2.3^a^
Aroclor_25_	16 ± 3.1^b^
Aroclor_50_	25 ± 5.2^c^

**Table 3:** *Toxic Effect of Progenitor Cell-Derived Condtioned Medium on Neutrophil Cultures.* Isolated neurophils (2 X 10^6^ cells/mL) were incubated with CMPC-Aroclor_(6, 12, 25 or 50 μM)_ or vehicle for 24 hrs. After this incubation, neutrophils were washed and viability was assessed by spectrophotometric analysis of LDH release and eosin Y uptake. Aroclor_(X)_ = CMPC-Aroclor_(X)_ = conditioned mediuM from PC treated with Aroclor, where (X) is the concentration of Aroclor; Values represent mean ± S.E.M.; a, indicates values that are significant (p≤0.05).

*Additions to Neutrophil Cultures*	*Neutrophil Viability (%)*
Naive	90 ± 7.4^a^
Vehicle	94 ± 3.5^a^
CMPC-Aroclor_6_	88 ± 5.6^a^
CMPC-Aroclor_12_	93 ± 9.1^a^
CMPC-Aroclor_25_	89 ± 8.3^a^
CMPC-Aroclor_50_	91 ± 7.4^a^

**Table 4:** *Chemotaxis of Neutrophils to Aroclor or Conditioned Medium from PC Treated with Aroclor.* In a two-chambered chemotaxis system, CMPC-Aroclor_(6, 12, 25, or 50 μM)_ was placed in the lower chamber and neutrophils (1 X 10^6^ cells) were placed in the upper chamber. After a 1hr incubation period, chemotaxis was determined by the nuMber of neutrophils that had migrated through the membrane pores to the lower chamber. Migration was expressed as % of positive control (FMLP). Aroclor_(X)_ = PCB mixture, Aroclor, where (X) is the concentration of Aroclor; CMPC-Aroclor_(X)_ = conditioned mediuM from PC treated with Aroclor, where (X) is the concentration of Aroclor; Values represent mean ± S.E.M.; a, b indicate values that are significantly different (p≤0.05).

*Chemotaxins*	*Neutrophil Chemotaxis (%FMLP)*
Naive	9 ± 3.3^a^
Vehicle	8 ± 3.2^a^
Aroclor_6_	7 ± 5.7^a^
Aroclor_12_	10 ± 7.4^a^
Aroclor_25_	7 ± 4.3^a^
Aroclor_50_	9 ± 6.1^a^
CMPC-Aroclor_6_	10 ± 4.4^a^
CMPC-Aroclor_12_	7 ± 3.1^a^
CMPC-Aroclor_25_	94 ± 5.1^b^
CMPC-Aroclor_50_	99 ± 7.2^b^

**Table 5:** *Induction of Neutrophil-Derived Protease Release by Conditioned Medium from PC Treated with Aroclor*. Isolated neurophils (2 X 10^6^ cells/mL) were incubated with CMPC-Aroclor _(6, 12, 25 or 50 μM)_ or vehicle for 24 hrs. After this incubation, culture mediuM as analyzed (spectrophotometry) for the content of cathepsin G and elastase. CMPC-Aroclor_(X)_ = conditioned mediuM from PC treated with Aroclor, where (X) is the concentration of Aroclor; Values represent mean ± S.E.M.; a, b, c indicate values that are significantly different (p≤0.05).

*Additions to Neutrophil Cultures*	*Release of the Protease Cathepsin G (U/L)*	*Release of the Protease Elastase (U/L)*
Naive	4 ± 2.2^a^	4 ± 1.2^a^
Vehicle	3 ± 3.1^a^	4 ± 2.3^a^
CMPC-Aroclor_6_	3 ± 2.4^a^	3 ± 1.5^a^
CMPC-Aroclor_12_	4 ± 1.5^a^	3 ± 2.1^a^
CMPC-Aroclor_25_	12 ± 2.3^b^	15 ± 2.6^b^
CMPC-Aroclor_50_	18 ± 2.2^c^	24 ± 3.3^b^

**Table 6:** *Induction of Neutrophil-Derived Super Oxide Release by Conditioned Medium from PC Treated with Aroclor.* Isolated neurophils (2 X 10^6^ cells/mL) were incubated with CMPC-Aroclor _(6, 12, 25 or 50 μM)_ or vehicle for 24 hrs. After this incubation, neutrophil-derived sueroxide production was analyzed using cytochrome C reduction. CMPC-Aroclor_(X)_ = conditioned mediuM from PC treated with Aroclor, where (X) is the concentration of Aroclor; Values represent mean ± S.E.M.; a, b indicate values that are significantly different (p≤0.05).

*Additions to Neutrophil Cultures*	*Neutrophil Superoxide Release (% FMLP)*
Naive	10 ± 3.3^a^
Vehicle	8 ± 5.7^a^
CMPC-Aroclor_6_	11 ± 3.2^a^
CMPC-Aroclor_12_	9 ± 1.2^a^
CMPC-Aroclor_25_	82 ± 5.5^b^
CMPC-Aroclor_50_	89 ± 4.2^b^

**Table 7:** *Induction of Neutrophil-Dependant PC toxicity by Conditioned Medium from PC Treated with Aroclor.* CMPC-Aroclor _(6, 12, 25 or 50 μM)_ or vehicle was administered to neutrophil/PC co-cultures (2 X 10^6^/5 × 10^6^ cells/mL, respectively) and incubated for 24 hrs. After this incubation, the content of hyaluronan in culture mediuM was analyzed as a marker of PC toxicity. CMPC-Aroclor_(X)_ = conditioned mediuM from PC treated with Aroclor, where (X) is the concentration of Aroclor; Values represent mean ± S.E.M.; a, b indicate values that are significantly different (p≤0.05).

*Additions to Progenitor Cell Cultures*	*Release of Hyaluronin (ng/ml) by Progenitor Cells*
Naïve	8 ± 5.1^a^
CMPC-Aroclor_6_	7 ± 4.3^a^
CMPC-Aroclor_12_	9 ± 4.4^a^
CMPC-Aroclor_25_	8 ± 1.9^a^
CMPC-Aroclor_50_	7 ± 3.8^a^
Neutrophils	8 ± 2.0^a^
CMPC-Aroclor_6_ + Neutrophils	8 ± 3.4^a^
CMPC-Aroclor_12_ + Neutrophils	7 ± 4.5^a^
CMPC-Aroclor_25_ + Neutrophils	158 ± 2.5^b^
CMPC-Aroclor_50_ + Neutrophils	162 ± 10.3^b^

**Table 8:** *Protease Inhibitor and Super Oxide Dismutase Affords Protection against Neutrophil-Dependant PC Toxicity*. Trypsin-chymotrypsin inhibitor (50 μg/well) and superoxide dismutase were added to neutrophil/PC co-cultures (2 X 10^6^/5 × 10^6^ cells/mL, respectively) treated with CMPC-Aroclor_(6, 12, 25 or 50 μM)_ or vehicle. Co-cultures were then incubated for 24 hrs. After this incubation, the content of hyaluronan in culture mediuM was analyzed as a marker of PC toxicity. CMPC-Aroclor_(X)_ = conditioned mediuM from PC treated with Aroclor, where (X) is the concentration of Aroclor; TCI = trypsin-chymotrypsin inhibitor; SOD = superoxide dismutase; Values represent mean ± S.E.M.; a, b, c indicate values that are significantly different (p≤0.05).

*Additions to Progenitor Cell Cultures*	*Release of Hyaluronan (ng/ml) by Progenitor Cells*
Naïve	8 ± 5.1^a^
TCI / SOD	7 ± 4.3^a^
Neutrophils	8 ± 2.0^a^
CMPC	8 ± 3.4^a^
CMPC-Aroclor_50_ + Neutrophils	162 ± 10.3^b^
CMPC-Aroclor_50_ + Neutrophils + TCI	78 ± 4.5^c^
CMPC-Aroclor_50_ + Neutrophils + SOD	69 ± 7.5^c^
CMPC-Aroclor_50_ + Neutrophils + TCI/SOD	11 ± 4.3^c^
